# Facile In Situ Synthesis of Supported Gold Catalysts on Ti_3_AlC_2_-Based Nanofibers for Selective Oxidation of Biomass-Derived HMF and Aromatic Alcohols

**DOI:** 10.3390/ma19194179

**Published:** 2026-09-30

**Authors:** Yicheng Sun, Jun Chen, Jun Yan, Xingguang Zhang

**Affiliations:** 1 YK PAO School, 1800 Lane 900 North Sanxin Road, Songjiang District, Shanghai 201620, China; 2 School of Materials and Chemistry, University of Shanghai for Science and Technology, 516 Jungong Road, Shanghai 200093, China; 3 College of Chemical Engineering, Nanjing Forestry University, Nanjing 210037, China

**Keywords:** gold catalyst preparation, Ti_3_AlC_2_ MAX phase, HMF, aromatic alcohols

## Abstract

Supported metal catalysts play a significant role in the selective organic synthesis and valorization of bio-derived platform molecules, and the development of catalyst preparation method is an important research topic. The facile in situ reduction synthesis strategy was investigated to prepare gold catalysts on Ti_3_AlC_2_-derived nanofibrous materials, using “as-produced H_2_” during hydrothermal synthesis as a reductive agent. The results of catalyst characterizations revealed that the metallic Au NPs (gold nanoparticles) were loaded with high efficiency. The new series of Au_pre_/Ti_3_Al_x_C_2_T_y_ catalysts as prepared by the in situ reduction method generally exhibited higher conversions in the selective oxidation of biomass-derived HMF (5-hydroxymethylfurfural) and aromatic alcohols, outperforming the traditional series of Au_dp_/Ti_3_Al_x_C_2_T_y_ catalysts as made by the deposition–precipitation method. The HMF conversion was 96.9% over 2%Au_pre_/Ti_3_Al_x_C_2_T_y_ and was 76.9% over 2%Au_dp_/Ti_3_Al_x_C_2_T_y_. The HMFCA (5-hydroxymethyl-2-furan carboxylic acid) selectivity over these two catalysts was almost identical, whereas it was 96.3% over 1%Au_pre_/Ti_3_Al_x_C_2_T_y_ and 84.3% over 1%Au_dp_/Ti_3_Al_x_C_2_T_y_. The catalyst reusability tests proved that the typical catalyst Au_pre_/Ti_3_Al_x_C_2_T_y_ maintained reaction rates and product selectivity in at least three cycles, whereas the reusability of Au_dp_/Ti_3_Al_x_C_2_T_y_ catalyst was relatively weaker in both reactions. Finally, the plausible catalytic mechanism was discussed according to peer studies and the research results.

## 1. Introduction

The worldwide industrialization and exploitation of natural resources deteriorates the global environment and intensifies the energy crisis and burgeoning societal issues, calling for the utilization of sustainable resources and eco-friendly chemical technology [1,2]. Biomass is one type of abundant renewable resource and has been popularly regarded as an alternative to fossil resources to supply raw materials, transport biofuels, and clean chemicals. For example, HMF (5-hydroxymethylfurfural) can be produced from carbohydrates (e.g., sugar and cellulose) and serves as a promising platform molecule for valorization [3,4]. Hence, developing eco-friendly chemical technology of highly selective catalytic processes to reduce such as energy dependence and environmental burden has significantly attracted researchers’ attention.

Selective oxidation via heterogeneous catalysis is a promising approach to producing value-added chemicals in organic synthesis. With regard to HMF, the value-added derivatives can be obtained, such as HMFCA (5-hydroxymethyl-2-furan carboxylic acid), DFF (2,5-dimethyl-furan), FFCA (5-formyl-2-furan carboxylic acid), and FDCA (2, 5-furan dicarboxylic acid) [5,6]. Among those, HMFCA has a high chemical stability and low toxicity and serves as a raw precursor for bio-based polyester and coatings with antitumor activity and an interleukin-inhibiting tendency. For aromatical alcohols, their corresponding aromatic aldehydes are important intermediates for producing pharmaceuticals, pesticides, fragrances, dyes and drugs [7,8]. Therefore, developing highly efficient catalysts for the selective oxidation of biomass-derived molecules and aromatic alcohols to value-added ones is of great importance in the utilization of renewable biomass and aromatic alcohols.

Heterogeneous catalysts of supported metal nanoparticles (NPs) have been widely applied in chemical industries, energy storage and environmental remediation [9,10]. In the above-mentioned selective oxidation of bio-derived molecules of HMF and aromatic alcohols, the design of catalytic structures, the advance in catalyst preparation technology, and the understanding of the catalytic mechanism are imperative in academic and engineering research [11,12]. Conventional technologies to prepare supported metal catalysts include the following methods: (1) the impregnation or deposition–precipitation along with chemical reduction process, utilizing sodium borohydride, formaldehyde, and hydrogen [13,14]; (2) the structure–reactivity construction strategy, employing the strong metal–support interaction (SMSI) [15,16], the electronic metal–support interaction (EMSI) [17,18], the reactive metal–support interaction (RMSI) [19]; and (3) single atomic (SA) synthesis or atomic layer deposition (ALD) [20,21]. Considering cost-effectiveness, metal loading efficiency and stability, a new family of catalytic materials and a preparation method are still needed to be further developed.

The catalyst supports mainly include carbon-based materials, zeolites, and metal oxides. MAX phases materials feature multilayered ternary metal carbides, nitrides or carbonitrides, and it has a general formula of M*_n_*_+1_AX*_n_* (M = early transition metal, A = A group elements, X = C or N, and *n* = 1–3) [22,23]. Ti_3_AlC_2_ is a representative and has been transformed into MXenes as reported in Gogotsi and Barsoum’s pioneered work in 2011 [24]. Numerous studies have focused on the electrical, thermal and mechanical properties and structural evolution of Ti_3_AlC_2_ to MXenes (e.g., Ti_3_C_2_ and Ti_2_C) and other nanomaterials or composites [25,26], and few studies concentrate on catalytic performances. Our recent study reported that when Ti_3_AlC_2_ was etched by an aqueous solution of sodium hydroxide under hydrothermal conditions (150–250 °C), solid products could be nanosheets, nanofibers, nanobelts or nanoparticles [27]. Other associated research reported that the following chemical reactions occurred when Ti_3_AlC_2_ was etched by NaOH solution [28,29]: (1) Ti_3_AlC_2_ + (1 − *x + y*) NaOH + (1 − *x*) H_2_O = Ti_3_Al_x_C_2_(ONa)*_y_* + (1 − *x*) NaAlO_2_ + 0.5 (3 − 3*x + y*) H_2_; (2) Ti_3_AlC_2_ + (1 − *x*) NaOH + (1 − *x + z*) H_2_O = Ti_3_Al_x_C_2_(OH)*_z_* + (1 − *x*) NaAlO_2_ + 0.5 (3 − 3*x + z*) H_2_, where 0 ≤ *x* < 1, 1 ≤ *y* or *z* < 2. Interestingly, the as-produced H_2_ can in situ reduce metal cationic precursors to metal NPs, which consequently are loaded on Ti_3_AlC_2_-derived nanofibers (denoted as Ti_3_Al_x_C_2_T_y_, T = -ONa, -OH or =O, and 0 < *x* < 1, 1 < *y* < 2). Our work reveals that the series of Pt/Ti_3_Al_x_C_2_T_y_ catalysts prepared by the in situ reduction method exhibit higher activity and selectivity in the selective hydrogenation of furfural and phenol, and also higher stability, than catalysts prepared by the traditional deposition–precipitation method [30,31].

Inspired by the above-mentioned studies, two series of Au catalysts were newly developed in this work (*m*%Au_pre_/Ti_3_Al_x_C_2_T_y_ by in situ reduction and *m*%Au_dp_/Ti_3_Al_x_C_2_T_y_ by deposition–precipitation, *m* = 0.5, 1.0, 1.5, 2.0). These catalysts were comprehensively evaluated in the selective oxidation of HMF and aromatic alcohols. The new series of Au_pre_/Ti_3_Al_x_C_2_T_y_ catalysts generally improved catalytic conversions in the two model reactions, as compared with those over the traditional series of Au_dp_/Ti_3_Al_x_C_2_T_y_ catalysts. In detail, the conversion of HMF was 96.9% over 2%Au_pre_/Ti_3_Al*_x_*C_2_T*_y_* and was 76.9% over 2%Au_dp_/Ti_3_Al*_x_*C_2_T*_y_*; the selectivity of HMFCA over these two catalysts was almost identical, whereas it was 96.3% over 1%Au_pre_/Ti_3_Al_x_C_2_T_y_ and 84.3% over 1%Au_dp_/Ti_3_Al_x_C_2_T_y_. The results of elemental content analysis proved that the new in situ reduction method more effectively loaded Au NPs on Ti_3_Al_x_C_2_T_y_ supports. The reusability tests demonstrated that the typical Au_pre_/Ti_3_Al_x_C_2_T_y_ catalyst could maintain reaction activity and product selectivity in at least three cycles, whereas the stability of the Au_dp_/Ti_3_Al_x_C_2_T_y_ catalyst was relatively weaker in both reactions. The nanofibrous structures of these catalysts offer facile separation from the liquid reaction system, and these research findings are anticipated to encourage peer researchers in terms of catalyst design for selective organic conversions.

## 2. Materials and Methods

### 2.1. Materials

All reagents and chemicals were analytical reagents, obtained from commercial sources, and utilized as received unless otherwise stated. Ti_3_AlC_2_ (98%, Beijing Hutong Wangyi Commerce & Trade Co., Ltd., Beijing, China). Sodium hydroxide (NaOH, 96%) and sodium borohydride (NaBH_4_, 98%) were purchased from Sinopharm Chemical Reagent Co., Ltd., Shanghai, China. Chloroauric acid (HAuCl_4_, 55% Au) was purchased from Macklin Chemical Reagent Co., Ltd., Shanghai, China. Benzyl alcohol (98%, denoted as Ph-CH_2_OH), benzaldehyde (98%, denoted as Ph-CHO), toluene (98%), cinnamic alcohol (98%), 4-methylbenzyl alcohol (98%), and 4-methoxylbenzyl alcohol (98%) were purchased from Shanghai Titan Technology Co., Ltd., Shanghai, China). HMF (5-hydroxymethylfurfural, 99%), HMFCA (5-hydroxymethyl-2-furancarboxylic acid, 98%), DFF (2,5-diformylfuran, 98%), FFCA (5-formyl-2-furancarboxylic acid, 98%) and FDCA (2,5-furandicarboxylic acid, 98%) were purchased from Shanghai Aladdin Biochemical Technology Co., Ltd. (Shanghai, China).

### 2.2. Catalyst Preparation

The series of Au_pre_/Ti_3_Al_x_C_2_T_y_ were prepared by the in situ reduction method [27]. Briefly, 15 mL of 10 mol/L NaOH aqueous solution was employed to disperse 1 g of Ti_3_AlC_2_ powder (200 mesh), and the mixture was stirred for 1 h continuously at ambient conditions to obtain a homogenous suspension. Then, the desired amount of HAuCl_4_ aqueous solution with preset concentration was added slowly with continuous stirring for another 1 h. Subsequently, the suspension was transferred into an autoclave (100 mL) with a PTFE container inside and it was kept at 180 °C for 48 h under static conditions in an oven. The final solid products were collected by filtration, along with thorough washing with deionized water until the pH value of the residual solution reached 7–9. The nominal Au loading of 0.25 wt%, 0.5 wt%, 1.0 wt%, 1.5 wt% and 2.0 wt% was obtained by changing the amount of HAuCl_4_ aqueous solution. The supports of Ti_3_Al_x_C_2_T_y_ were pre-prepared under identical conditions without Au loading.

The series of Au_dp_/Ti_3_Al_x_C_2_T_y_ catalysts were prepared for comparison by the conventional deposition–precipitation (DP) method [32]. Typically, in a glass beaker with a magnetic stirring bar, a certain amount of aqueous solution of HAuCl_4_ (1.0 g of HAuCl_4_ dissolved in 100 mL water) was utilized to disperse 2.5 g of Ti_3_Al_x_C_2_T_y_ supports to achieve the nominal loading of Au (e.g., 2% wt). 20 mL of 0.53 mol/L lysine aqueous solution was subsequently added dropwise to the suspension under continuous magnetic stirring for 0.5 h. Then, 10 mL of 0.35 mol/L NaBH_4_ aqueous solution was added dropwise, followed by adding 10 mL of 0.3 mol/L HCl aqueous solution to achieve a pH of around 9.5. The mixture was further stirred for 1 h and then aged for 24 h. The final solid products were separated by filtration and washed firstly with sufficient deionized water three times to remove the chlorine ions. The residual solution was tested by 0.1 mol/L AgNO_3_ aqueous solution to ensure that no AgCl precipitate was observed.

### 2.3. Characterizations

The crystal phases of samples and the crystallinity of materials were examined by X-ray diffraction (XRD), utilizing the diffractometer Rigaku Smartlab (Rigaku Corporation, Akishima, Tokyo, Japan) with the Cu Kα radiation (λ = 0.1542 nm) at the voltage of 40 kV and at the scan rate of 5°/min from 5 to 80 (2θ). The morphological information was examined by scanning electron microscopy (SEM) on the equipment of JSM-7600F (JEOL Ltd., Akishima, Japan) with an operating voltage of 30 kV. The morphologies of the target catalysts and the diameters of Au NPs were also examined by transmission electron microscopy (TEM) on the JEOL JEM-2100 (JEOL Ltd., Akishima, Japan) apparatus with the working voltage being 200 kV, and the sizes of around 200 particles were collected to calculate the average diameter. The chemical states of key elements were detected by X-ray photoelectron spectroscopy (XPS) on the Kratos Axis HSi X-ray photoelectron spectrometer (Kratos Analytical, Manchester, UK); it was fitted with a charge neutralizer and magnetic focusing lens, employing Al Kα monochromatic radiation (1486.7 eV). The software of CasaXPS version 2.3.16 was employed for fitting spectra. The binding energy was corrected using the C 1s peak at 284.8 eV, and the surface elemental proportions (surface atomic compositions) were calculated via correction for the appropriate instrument response factors. The spectra of Au 4f were fitted using a Doniac Sunjic modified Gaussian–Lorentzian asymmetric line shape. The practical loading Au was analyzed by the Inductive Coupled Plasma (ICP) on the NeXion 300X (PerkinElmer, Inc., Shelton, CT, USA) instrument. Typically, 20 mg of catalyst was digested with 10 mL of freshly prepared aqua regia in a PTFE container, which subsequently was ultrasonicated at room-temperature for at least 4 h, and followed by dilution and filtration before measurement.

### 2.4. Catalytic Reactions

**Selective oxidation of HMF**: The reactions were conducted on the basis of previous studies [5,33]. The blank sample was taken, using a syringe to aspirate 1.5 mL of reaction solution, and subsequently the liquid was filtered through a porous filter (0.22 μm). The filter head was saturated by 0.5 mL of solution, the residual 1.0 mL was filtered into a GC glass bottle (sample t_0_). Briefly, 50 mg of catalysts were dispersed in 10 mL of 0.1 mol/L HMF aqueous solution containing Na_2_CO_3_. The mixture was stirred at room temperature for 2 h to rule out the adsorption effect, and another sample was taken using the same syringe and saturated filter (sample t_2_). The above suspension was transferred into the autoclave reactor and it was sealed and purged with pure oxygen (O_2_) three times to remove air. Then, it was pressurized to 0.5 MPa with O_2_ and heated to 120 °C under stirring at 600 rpm. After reaction for 5 h, the autoclave was naturally cooled down to room temperature and then the inner residual gas was discharged. Finally, 1.5 mL of liquid sample was withdrawn using the same syringe and saturated filter (sample t_5_) to remove solid catalysts.

The liquid samples were analyzed on the HPLC (high-performance liquid chromatography, EClassical 3100, Dalian Elite Analytical Instruments Co., Ltd., Dalian, China) using the ChromCore Sugar-10H column (Dalian Elite Analytical Instruments Co., Ltd., Dalian, China) whose temperature was maintained at 30 °C. The flow rate of mobile phase (H_2_SO_4_ solution, 5 mmol/L) was 0.5 mL/min, and the detection wavelength was 260 nm on a UV detector. Standard curves of HMF, HMFCA, FFCA, and FDCA were performed for quantification, and the response factors were determined by respective multipoint calibration curves. The conversion, selectivity and the reaction rate were calculated using the following equations (*t* = 0 referred to a sample of solution being taken before reaction; *t* = *i* referred to a sample being taken after *i* h reaction. The reactant was HMF or benzyl alcohol; the product was HMFCA or benzaldehyde):

(1) $$\mathrm{Conversion}=\frac{{\mathrm{moles}\;\mathrm{of}\;\mathrm{reactant}}_{t=0}-{\mathrm{moles}\;\mathrm{of}\;\mathrm{reactant}}_{t=i}}{{\mathrm{moles}\;\mathrm{of}\;\mathrm{reactant}}_{t=0}}\times 100\%$$

(2) $$\mathrm{Selectivity}=\frac{{\mathrm{moles}\;\mathrm{of}\;\mathrm{product}}_{t=i}}{{\mathrm{moles}\;\mathrm{of}\;\mathrm{reactant}}_{t=0}-{\mathrm{moles}\;\mathrm{of}\;\mathrm{reactant}}_{t=i}}\times 100\%$$

Au NPs were catalytic centers of catalysts; the terminal reaction rate (mmol_reactant_·g_Au_^−1^·h^−1^) was calculated according to the following equation on the basis of the real loading of Au content detected by ICP after *i* h reaction:

(3) $$\mathrm{Reaction}\;\mathrm{rate}=\frac{\mathrm{Moles}\;\mathrm{of}\;\mathrm{reactant}\;\mathrm{converted}}{\mathrm{Gram}\;\mathrm{of}\;\mathrm{gold}\;\mathrm{loading}\times \mathrm{Reaction}\;\mathrm{time}}$$

**Selective oxidation of benzyl alcohol**: The catalysts were evaluated in the selective aerobic oxidation of Ph-CH_2_OH and the experimental setup was on the basis our recent study [8]. The reaction was carried out in a 50 mL round-bottomed Pyrex glass flask which was sealed by a rubber spigot. In a typical procedure, 20 mL of toluene as solvent was used to dissolve 3.5 mmol of Ph-CH_2_OH, 0.35 mmol of NaOH (molar ratio 0.35/3.5 = 10%) under stirring to obtain a homogeneous reaction solution. Then 200 mg of catalysts were added into the solution, and subsequently the suspension was continuously stirred for 2 h to rule out the adsorption effect, then a control sample was collected. For sampling, 1 mL aliquots were aspirated using a syringe and filtered through a Millipore filter (pore size 0.22 μm) to remove the catalysts powders and to saturate the filter head. Then, another 1 mL of reaction solution was aspirated and filtered using the same filter. After sampling, the glass flask with was purged with O_2_ for three minutes to replace the air and then sealed, and the reactor was submerged into the oil bath which had been pre-heated to 90 °C. After reaction for 4 h, the sample was obtained by the above syringe. For other aromatic alcohols, the test was performed under identical conditions.

The detection and quantification were conducted on a Gas Chromatograph (GC 9790H, produced by Zhejiang FULI, Wenling, China) which was equipped with the PEG-20M column. For quantification, the analysis of each sample was performed in triplicate, with a peak area reproducibility of ±2% for standards such as Ph-CH_2_OH and Ph-CHO, and ±10% for those samples if the concentration of product was extremely low. Response factors for each aromatic alcohol and the corresponding aldehyde were determined from respective multipoint calibration curves. The conversion and reaction rate of aromatic alcohols and the product selectivity were calculated according to the above three Equations: (1)–(3).

## 3. Results and Discussion

### 3.1. Crystal Structures of Catalysts by XRD

**Figure 1:**
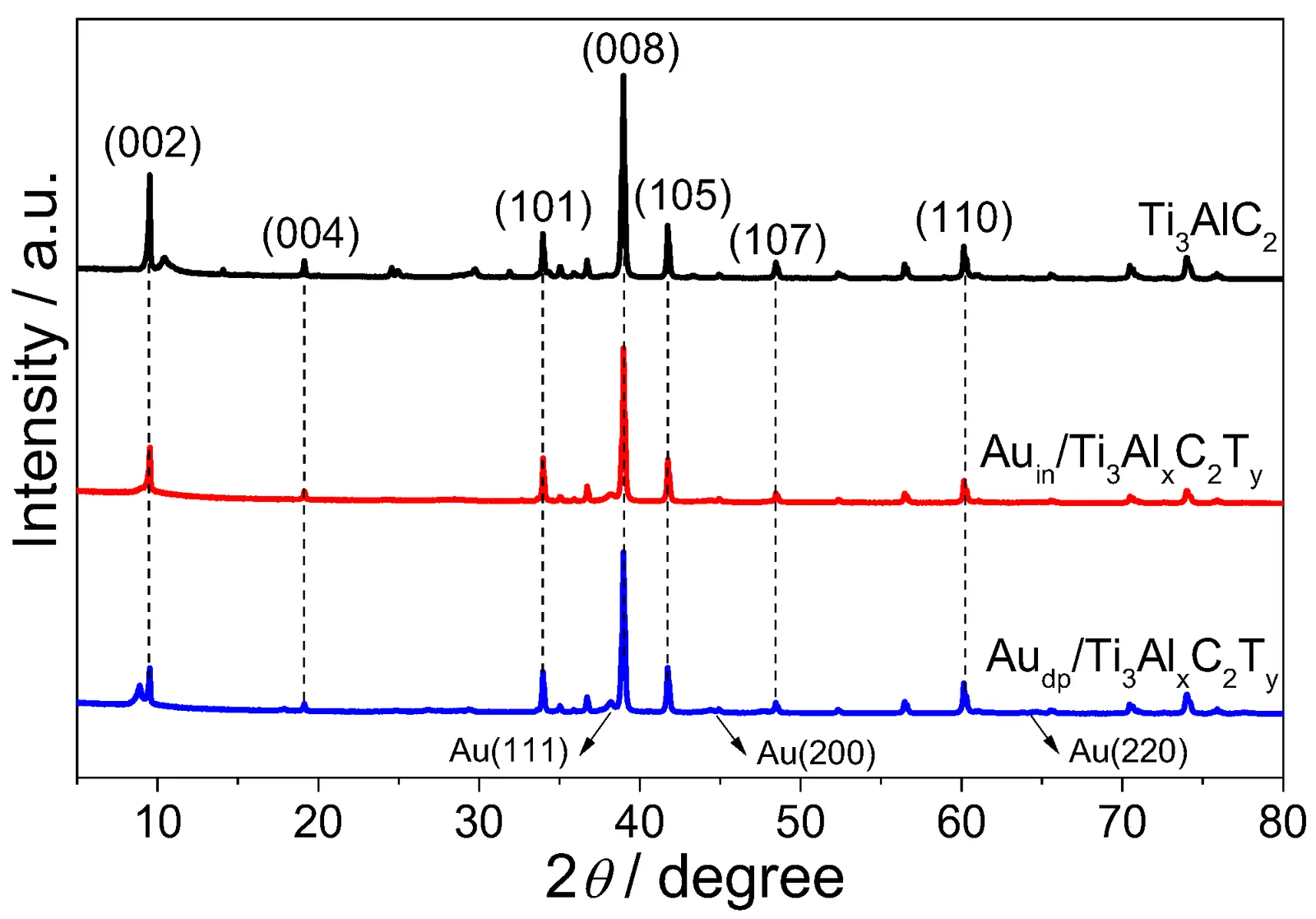
The XRD patterns of the original Ti_3_AlC_2_ MAX phase, 2%Au_pre_/Ti_3_Al_x_C_2_T_y_, and 2%Au_dp_/Ti_3_Al_x_C_2_T_y_, (more XRD patterns of two series of catalysts were provided in Figure S1, ESI).

The crystal phases and crystallinity were characterized by XRD technology; their patterns were shown in Figure 1. Compared with the XRD pattern of original Ti_3_AlC_2_, the two typical catalysts of 2%Au_pre_/Ti_3_Al_x_C_2_T_y_ and 2%Au_dp_/Ti_3_Al_x_C_2_T_y_ also exhibited the featured diffraction peaks at 9.5°, 19.1°, 36.7°, 39.0°, 41.7°, 48.4° and 60.1°; they were assigned to the crystal planes of (002), (004), (101), (008), (105), (107) and (110), corresponding to the typical crystal phases of Ti_3_AlC_2_ (JCPDF no. 52-0875) [34]. Except for the slight difference in intensity at the two theta angle of 9.5° (002), these results suggested that the as-made Ti_3_Al_x_C_2_T_y_ supports maintained the primary crystal structures of Ti_3_AlC_2_ after loading of Au NPs and the removal of Al element by etching without major structural changes. These results are consistent with Ng and co-workers’ observations [35].

The intensities of XRD peaks on 2%Au_pre_/Ti_3_Al_x_C_2_T_y_ and 2%Au_dp_/Ti_3_Al_x_C_2_T_y_ were slightly weaker than those of Ti_3_AlC_2_ after the removal of Al and thus resulted in a slight decline in crystallinity. For instance, the typical peak (008) at ∼39° confirmed the multilayer samples containing residual and unetched Ti_3_AlC_2_ MAX phase. The minorly altered peak of (002) was due to the formation of surface Ti_3_C_2_(OH)_2_, which confirmed the removal of surface Al by NaOH [36]. To explain, the Al component in Ti_3_AlC_2_ dissolved in NaOH as soluble Al(OH)_4_^−^ and exposed Ti and Al atoms which terminated by -OH/Na or =O. In sufficient concentrated alkaline solution under heated hydrothermal conditions, continuous etching process could further remove Al atoms, and meanwhile inevitably generated Al hydroxides (e.g., (Al(OH)_3_) [35,36].

After loading of Au NPs, three peaks appeared at around 38.2°, 44.5° and 64.5° over 2%Au_pre_/Ti_3_Al_x_C_2_T_y_ and 2%Au_dp_/Ti_3_Al_x_C_2_T_y_, and they were the characteristic diffraction peaks of metallic Au NPs, corresponding to the crystal planes of Au (111) (38.2°), Au (200) (44.5°) and Au (220) (64.5°) [37]. These peaks revealed that Au NPs effectively loaded on Ti_3_Al_x_C_2_T_y_ supports.

### 3.2. SEM and TEM Analyses on Sizes and Morphologies

SEM and TEM analyses were performed to examine the morphology of two catalysts of 2%Au_pre_/Ti_3_Al_x_C_2_T_y_ and 2%Au_dp_/Ti_3_Al_x_C_2_T_y_, and the particle size distribution of Au NPs.

**Figure 2:**
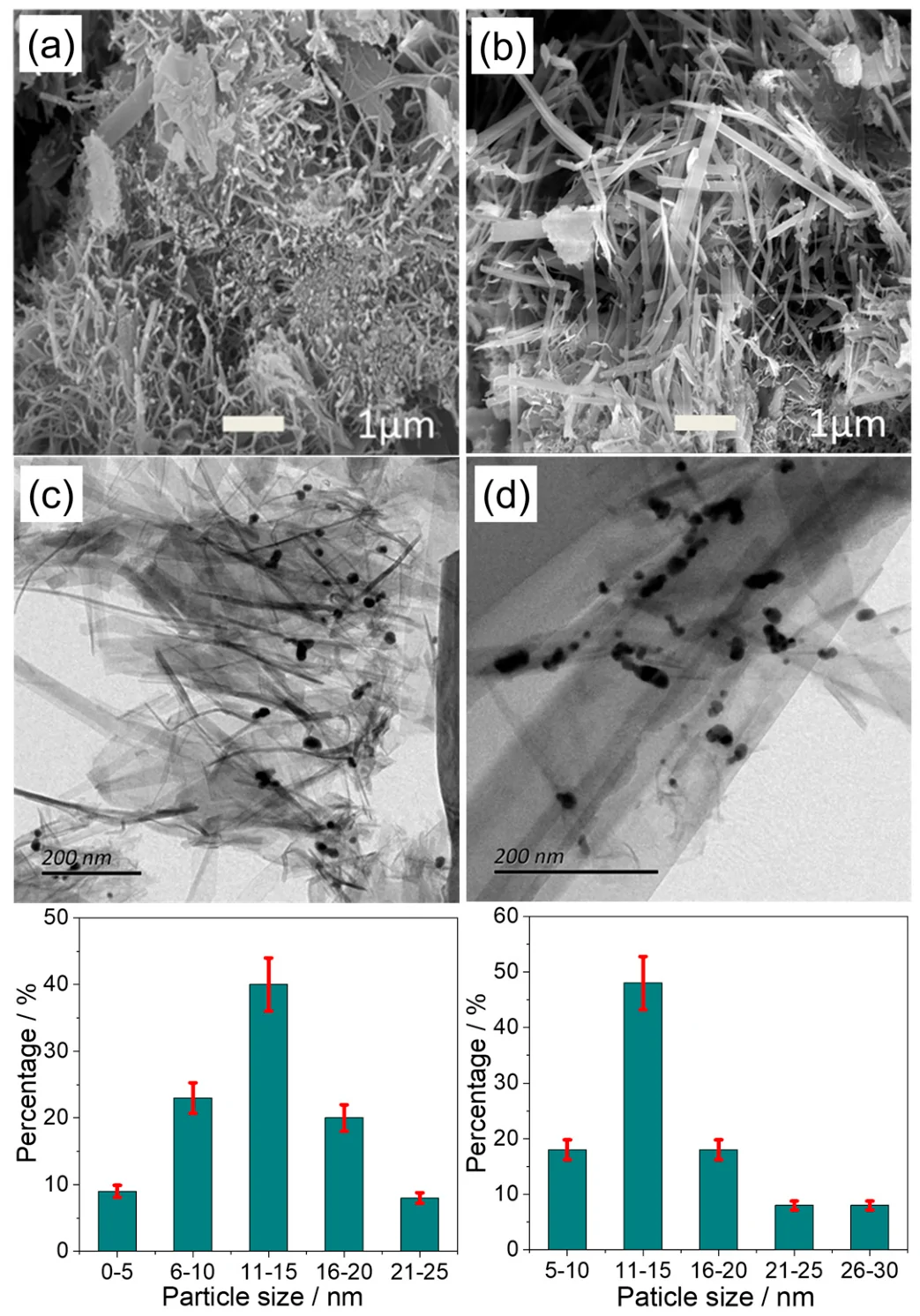
(**a**,**b**) SEM images and (**c**,**d**) TEM images and corresponding histograms of Au NPs‘ size distribution over two typical catalysts of Au_pre_/Ti_3_Al_x_C_2_T_y_ (**left panel**) and Au_dp_/Ti_3_Al_x_C_2_T_y_ (**right panel**). Error bars: 10%.

The SEM images (Figure 2a,b) and the TEM images (Figure 2c,d) presented that the catalysts were primarily nanofibrous materials; such structures are more advantageous than nanoparticles for recycling from liquid reaction systems. The TEM images further provided detailed information of size, shape and distribution of Au NPs on Ti_3_Al_x_C_2_T_y_, showing that Au NPs were pseudo-spherical nanoparticles and had a size distribution centering at approximately 14.7 nm. As displayed in the histogram, the average particle size of Au_pre_ NPs over 2%Au_pre_/Ti_3_Al_x_C_2_T_y_ (Figure 2c) was slightly smaller (11.2 nm) than that of 2%Au_dp_ NPs over Au_dp_/Ti_3_Al_x_C_2_T_y_ (Figure 2d).

### 3.3. XPS Analysis of Oxidation States

**Figure 3:**
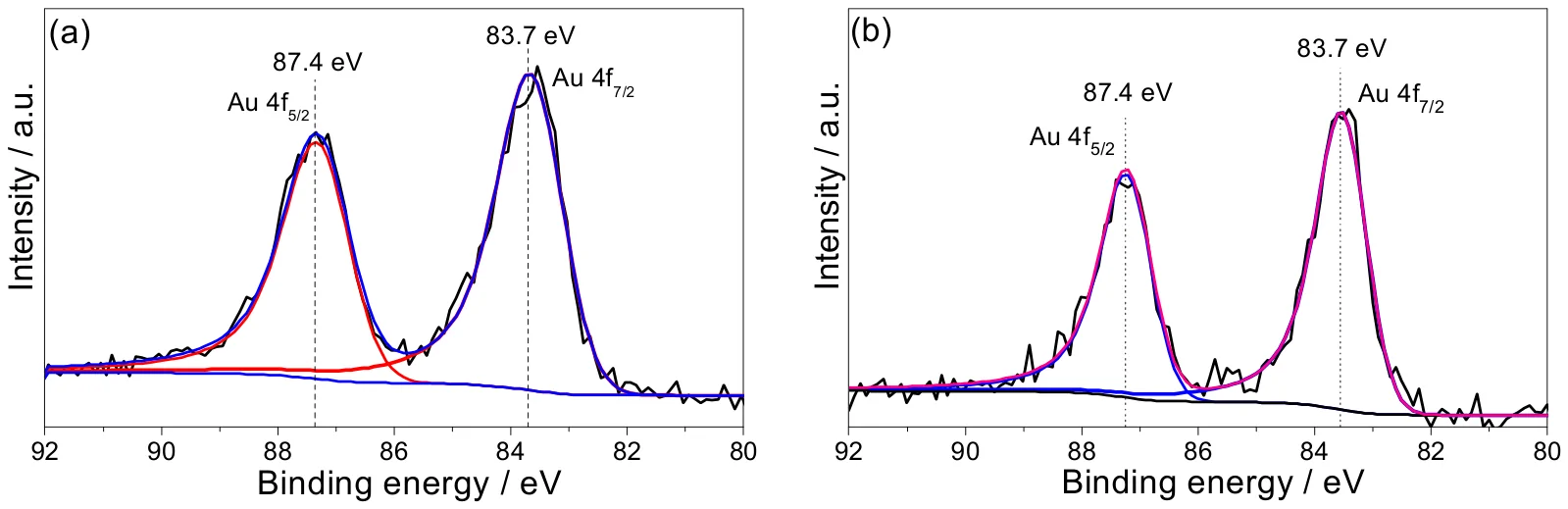
High-resolution XPS spectra of Au 4f over (**a**) Au_pre_/Ti_3_Al_x_C_2_T_y_ and (**b**) Au_dp_/Ti_3_Al_x_C_2_T_y._

One of the principal purposes of this study is to substantiate that metallic Au are formed on the catalysts of Au_pre_/Ti_3_Al_x_C_2_T_y_ under in situ reduction conditions. Hence, it is imperative to examine the chemical state of Au by detailed analyses of high-resolution XPS. Expectedly, metallic Au was achieved without using any extra reductive agents under the in situ reduction conditions, and the high-resolution XPS spectra of Au 4f was given in Figure 3a, showing the binding energies of 83.7 eV (Au 4f_7/2_) and 87.4 eV (Au 4f_5/2_) [38]. To further confirm the effectiveness of this in situ reduction method, the control catalyst of Au_dp_/Ti_3_Al_x_C_2_T_y_, as prepared via the traditional deposition–precipitation, was also examined by XPS analysis. The high-resolution XPS spectra of Au 4f in Figure 3b proved that the binding energies were 83.7 eV (Au 4f_7/2_) and 87.4 eV (Au 4f_5/2_) on the control catalyst. Moreover, for both Au_pre_/Ti_3_Al_x_C_2_T_y_ and Au_dp_/Ti_3_Al_x_C_2_T_y_, negligible positively/negatively charged Au species were identified (see Table S1, ESI for more information on the binding energies of gold species of different oxidation states). These results demonstrated that the in situ reduction strategy was essentially as efficient as the traditional deposition–precipitation method, and it should be more cost-effective as H_2_ was released from the chemcial transformation of raw support materials of Ti_3_AlC_2_.

As to other key elements of C1s, O 1s, and Ti 2p, the high-resolution XPS spectra were also analyzed (Figure S2, ESI). These XPS analyses offered critical information on the surface species of Au_pre_/Ti_3_Al_x_C_2_T_y_ and confirmed the existence of C-C, C-O/C=O [39], the lattice O (Ti-O-Ti), the bridging O (Ti=O), the top-site O (Ti-OH/Na) [40], two major spin-orbital peaks of Ti^4+^ and associated sub-stoichiometric titanium carbides (TiC_x_, x < 1) or titanium oxycarbides (O-Ti-C) [41]. Overall, the noise of Au 4f spectra was stronger than that of C 1s, O 1s, and Ti 2p spectra, which was principally ascribed to the relatively low content of Au which resulted in a low photoelectron count rate (namely weaker signal), as compared with those elements in the catalyst samples.

### 3.4. Effect of Gold Content on Selective Oxidation

**Figure 4:**
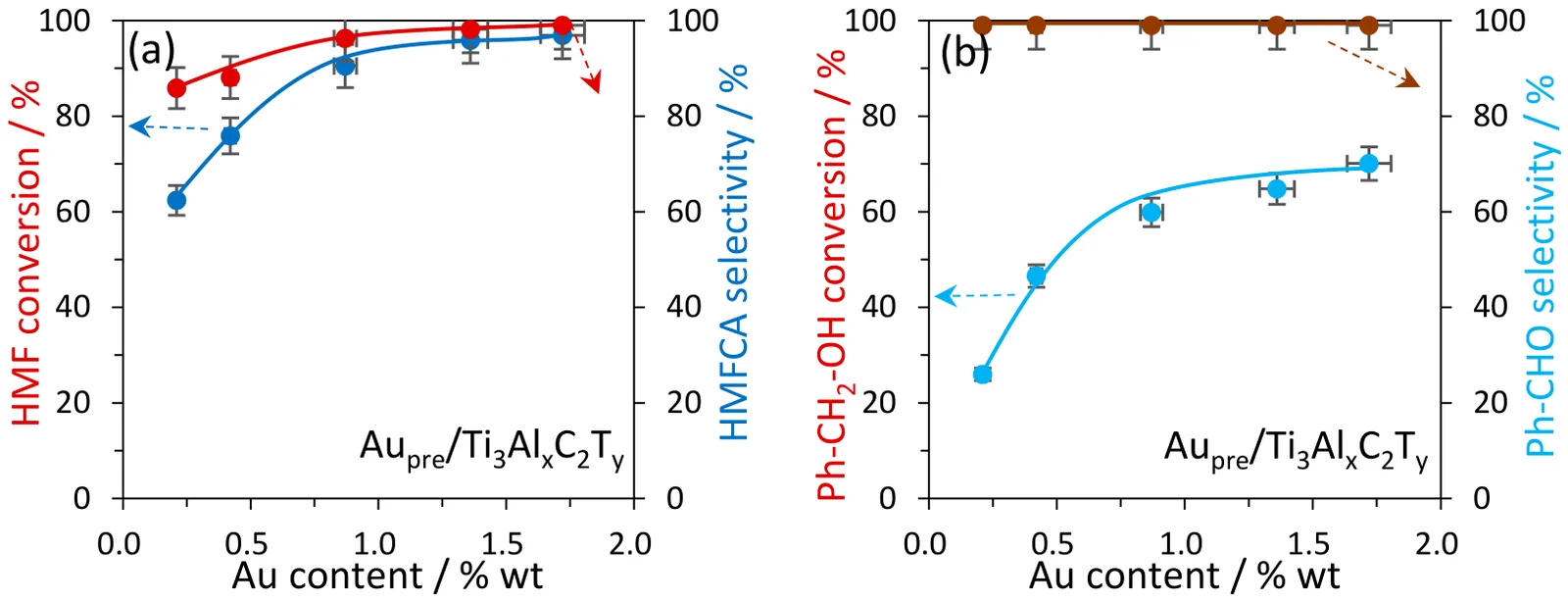
Catalytic performances in selective oxidation of (**a**) HMF to HMFCA and (**b**) Ph-CH_2_OH to Ph-CHO over Au_pre_/Ti_3_Al_x_C_2_T_y_ as the function of real Au loading. **Reaction conditions:** HMF (reactant, 1 mmol), H_2_O (solvent, 10 mL), Na_2_CO_3_ (2 mmol), catalysts (50 mg), 120 °C, 5 h, 0.5 MPa O_2,_ 600 rpm. The reaction conditions for Ph-CH_2_OH was provided in Figure 5. Error bars: 5%.

**Figure 5:**
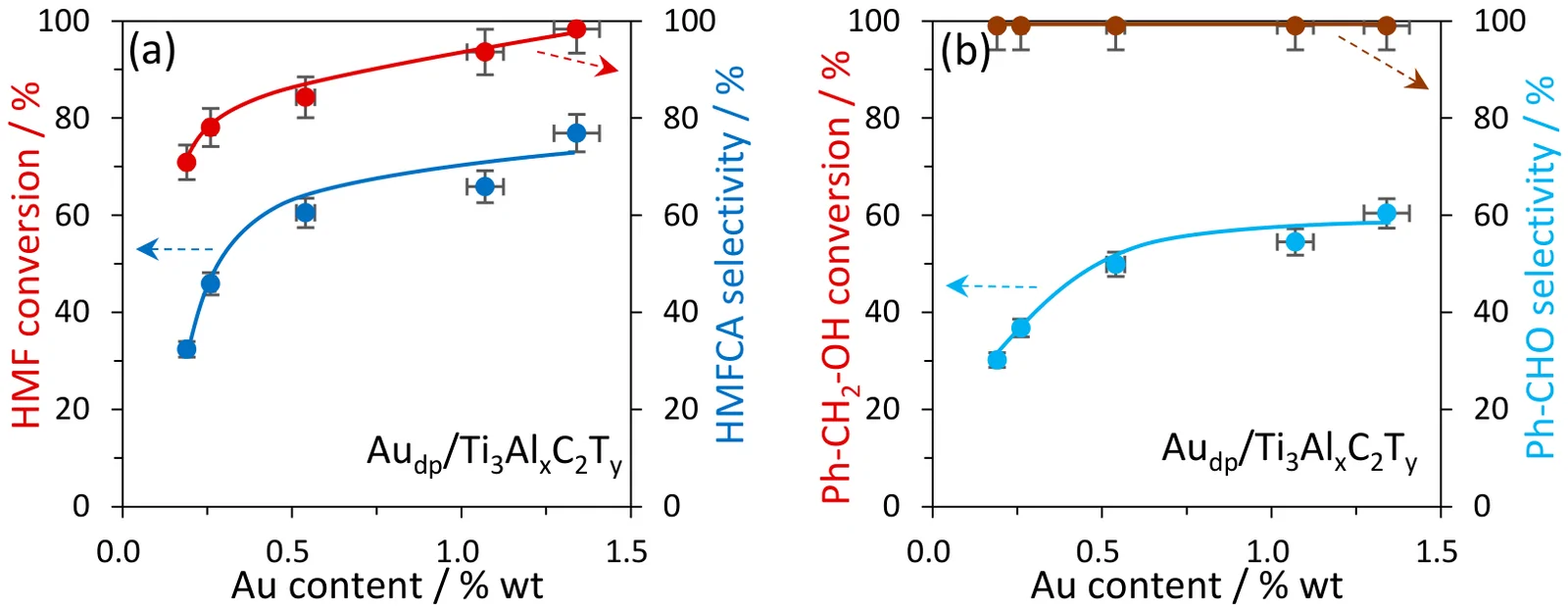
Catalytic performances in selective oxidation of (**a**) HMF and (**b**) Ph-CH_2_OH over Au_dp_/Ti_3_Al_x_C_2_T_y_ as the function of real Au loading. **Reaction conditions:** Ph-CH_2_OH (reactant, 3.5 mmol), sodium hydroxide (0.35 mmol), toluene (solvent, 20 mL), catalysts (200 mg), 90 °C, 4 h, O_2_ atmosphere. The reaction conditions for HMF was provided in Figure 5. Error bars: 5%.

The catalytic performances of two series of Au_pre_/Ti_3_Al_x_C_2_T_y_ and Au_dp_/Ti_3_Al_x_C_2_T_y_ catalysts with different Au contents were firstly evaluated in selective oxidation of HMF and the results of catalytic tests are shown in Figure 4a and Figure 5a. Above all, in the control test with Ti_3_Al_x_C_2_T_y_ supports only examined as catalysts, the HMF conversion was lower than 5.0%, owing to the homogeneous effect, and thus hydrochar species such as humins formed [27,42]. The negligible target product of HMFCA or other products were quantifiable, thereby confirming the essential catalytic contributions of Au as active sites.

As displayed in Figure 4a and Figure 5a, HMFCA was the primary product over Au-based catalysts of Au_pre_/Ti_3_Al_x_C_2_T_y_ and Au_pre_/Ti_3_Al_x_C_2_T_y_ under reaction conditions (a trace of DFF and FFCA were unquantifiable and FDCA was undetectable), and these observations were consistent to reported results in peer studies [5]. The gradual growing content of Au resulted in an boosted HMF conversion from 62.4% to 96.9% over Au_pre_/Ti_3_Al_x_C_2_T_y_ catalyst series and from 32.4% to 72.9% over Au_dp_/Ti_3_Al_x_C_2_T_y_. For both catalysts, the change in HMF conversions initially showed remarkable increase as the function of Au content from 0.25% to 0.5% (nominal loading). To explain, this phenomenon was due to more catalytic centers were accessible under certain initial concentration of reactant molecules (HMF and O_2_). Nevertheless, the HMF conversions exhibited not very rapid growth with continuous addition of Au content, potentially owing to the reduced diffusion rates of reactant molecules to active centers. The HMFCA selectivity also correspondingly improved from 85.9% to 99.0% over Au_pre_/Ti_3_Al_x_C_2_T_y_ catalyst series and from 70.9% to 98.3% over Au_dp_/Ti_3_Al_x_C_2_T_y_. When the Au loading was lower than 1.0%, the conversion and selectivity were relatively low, which was probably due to insufficient Au active sites to ensure selective oxidation reactions, and thus, the side reactions occurred. For instance, solid humins were formed and thus resulted in a small proportion of mass loss [43].

For the selective oxidation of alcohols with O_2_, the catalytic performances of Au_pre_/Ti_3_Al_x_C_2_T_y_ and Au_dp_/Ti_3_Al_x_C_2_T_y_ were compared under identical reaction conditions, employing selective oxidation of Ph-CH_2_OH to Ph-CHO as the model reaction at 90 °C [44]. Interestingly, the improving content of Au loadings hardly affected the selectivity toward Ph-CHO over both series of catalysts (>99%), as presented in Figure 4b and Figure 5b [45]. The catalytic conversions of Ph-CH_2_OH over Au_pre_/Ti_3_Al_x_C_2_T_y_ catalyst series generally outperformed those over Au_dp_/Ti_3_Al_x_C_2_T_y_ as the function of growing Au content. In detail, the conversion of Ph-CH_2_OH rose from 26.0% to 70.1% over Au_pre_/Ti_3_Al_x_C_2_T_y_ catalyst series and from 30.2% to 60.4% over Au_dp_/Ti_3_Al_x_C_2_T_y_ catalyst series. The reasons for the changing trendlines of Ph-CH_2_OH conversions were similar to those of HMF conversions. Namely, the initial remarkable change in conversions was controlled by the number of Au active centers, and the relatively steady state at higher Au loadings was due the slow diffusion rates of reactant moles to active sites.

Overall, for the two model reactions, the improvement in conversion over Au_pre_/Ti_3_Al_x_C_2_T_y_ catalysts could be due to the relative smaller sizes of Au NPs and higher real loading of Au content, as proved by the ICP results (Table S2, ESI), which also revealed the effectiveness of the in situ method to introduce Au NPs onto Ti_3_Al_x_C_2_T_y_. Moreover, the series of Au_pre_/Ti_3_Al_x_C_2_T_y_ catalysts exhibited higher reaction rates of HMF (mmol·g_Au_^−1^·h^−1^) than those over the series of Au_dp_/Ti_3_Al_x_C_2_T_y_ catalysts (Figure S3a, ESI); and the two series of catalysts generally showed slightly better reaction rates in the selective oxidation of Ph-CH_2_OH (Figure S3b, ESI). The above results further confirmed the advantages of new series of catalysts. The higher loading efficiency potentially resulted from the stronger interfacial interaction or adhesion between Au_pre_ NPs and Ti_3_Al_x_C_2_T_y_, which formed coherently during the in situ transformation and thus offered more interfacial sites to facilitate the electron transfer process, or exerted other synergistic effects [46,47]. For instance, Chen and co-workers prepared M-TiO_2_ catalysts (M = Au, Ag, Pt, or Pd) by an in situ photo-deposition method, and they found that the heterojunction of metal–support facilitated the transferring of electrons from metal to TiO_2_ [48]. In comparison, in the traditional deposition–precipitation method, the adhesion force between Au_dp_ NPs and pre-made Ti_3_Al_x_C_2_T_y_ was probably weaker and the interfacial connections were much fewer than those between Au_pre_ NPs, and Ti_3_Al_x_C_2_T_y_ formed in situ.

### 3.5. Influence of Substitutional Groups for Aromatic Alcohols

To test the general applicability of these newly developed catalysts, different aromatic alcohols with functional groups were examined over Au_pre_/Ti_3_Al_x_C_2_T_y_, the product selectivity was excellent with regard to the selected aromatic alcohols (>99%, in Table 1). For catalytic activity, these reactants exhibited “good to excellent” conversions, with 4-methoxybenzyl alcohol giving the highest conversion of 90.6%.

The reasons for the differences in conversions of these reactants have been investigated in many studies, involving electronic effects, steric effects, adsorptive energies, molecular polarities of reactants and the like [40]. For example, for the para-substituted reactants, the catalytic activity followed the Hammett rule; accordingly, substitution with electron-donating groups such as -OCH_3_ and -CH_3_ exhibited higher conversions [49]. In the current stage, it is challenging to quantify the individual contribution of each influential factor on the basis of experimental results; nonetheless, these results showed that Au_pre_/Ti_3_Al_x_C_2_T_y_ was a potential candidate for applications at a large scale because the fibrous catalysts are more facile to recycle from liquid catalytic systems compared with nanoparticulate catalysts.

### 3.6. Mechanistic Discussion

**Scheme 1:**
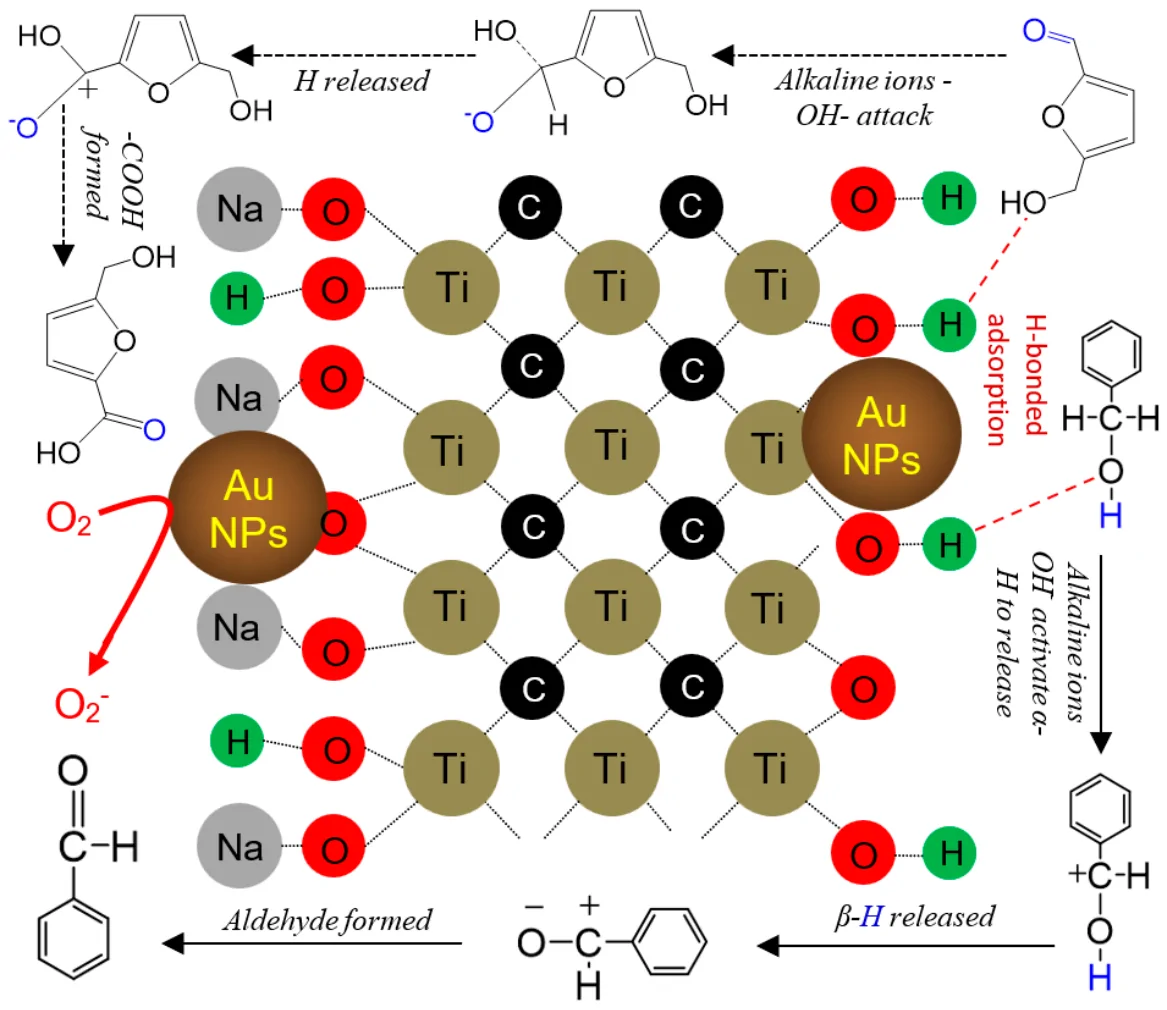
Proposed mechanism of selective oxidation HMF and Ph-CH_2_OH with molecular O_2_ over the typical catalyst of Au_pre_/Ti_3_Al_x_C_2_T_y._

Based on the heterogeneous catalytic mechanism of such as Langmuir–Hinshelwood (L-H) and other associated studies [50], the fundamental processes include three principal steps: (1) the adsorption of reactant molecules (e.g., O_2_, HMF and Ph-CH_2_OH) on catalyst surfaces (Au active sites, and/or Ti_3_Al_x_C_2_T_y_ supports), (2) the surface reactions between activated oxygen species and adsorbed intermediates, and (3) the desorption of products (e.g., HMFCA and Ph-CHO) from catalyst surfaces. The aforementioned XPS results on Au 4f confirmed the existence of metallic Au NPs, surface oxygen-containing species of Ti_3_Al_x_C_2_T_y_ supports, such as C-O/C=O [39], lattice O, bridging and top-site O [40], and titanium-associated species (e.g., sub-stoichiometric titanium carbides and titanium oxycarbides) [41]. The reactant molecules were potentially adsorbed on the surface of both Au NPs and Ti_3_Al_x_C_2_T_y_ supports. The adsorption force should be electronic interaction and/or hydrogen bonds [51]. As shown in Scheme 1, the adsorbed O_2_ molecules were activated on Au active centers to form reactive oxygen species (e.g., O_2_^−^) to collect H-species released from HMF or Ph-CH_2_OH to form side product of H_2_O.

As for HMF molecules, the adsorption effect concentrated them on catalyst surfaces by such as H-bonds and contributed to initializing the reactions. The presence of hydroxyl ions (OH^−^) derived from the Na_2_CO_3_ solution attacked the -CHO groups on HMF to assist nucleophilic addition to produce an alkoxy intermediate of geminal diol [52]. Subsequently, the alkoxy carbon atom of that intermediate released H-species to produce HMFCA [53]. Notably, without enough Au active sites, homogeneous reactions were inevitable to give side products (e.g., humins, hydrochar species) and thus severely declined HMFCA selectivity [54].

In the selective oxidation of aromatic alcohols to aldehydes, after the adsorption of reactant molecules by H-bonds, the pivotal reaction steps include the cleavage of O-H and α-C-H bonds to release H-species; peer studies elaborate the catalytic mechanisms and reaction pathways, which depend upon the catalyst categories [55], the chemical state of elements, surface functional groups or defects [56]. In this study, the alkaline additives (OH^-^) in the solution induces the methylene group (-CH_2_-) to combine with Au NPs to form an “Au-benzyl alcoholic intermediate”, so that the α-H of methylene group were activated by Au active centers and released from the intermediate. Subsequently, the benzyl alcoholic carbocation intermediates might automatically release protonic hydrogen (β-H) from hydroxyl groups (-OH) to form C=O groups. Then the final product of Ph-CHO desorbed from the catalysts, so that the Au active centers and supports were regenerated [57].

### 3.7. Reusability Tests

**Figure 6:**
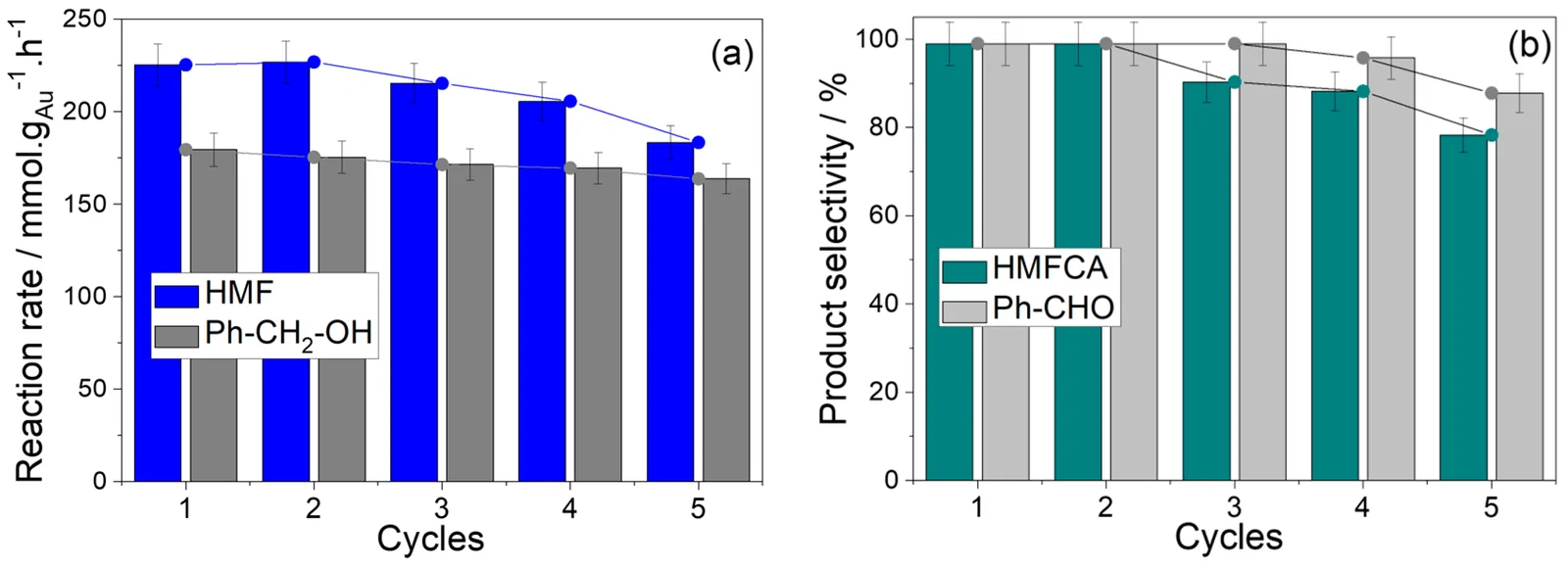
The reusability test of 2%Au_pre_/Ti_3_Al_x_C_2_T_y_ for selective oxidation of HMF and Ph-CH_2_OH: (**a**) reaction rate, and (**b**) selectivity of HMFCA or Ph-CHO. **Reaction conditions:** HMF (reactant, 1 mmol), H_2_O (solvent, 10 mL), Na_2_CO_3_ (2 mmol), catalyst (50 mg), 120 °C, 5 h, 0.5 MPa O_2,_ 600 rpm; and Ph-CH_2_OH (reactant, 3.5 mmol), sodium hydroxide (0.35 mmol), toluene (solvent, 20 mL), catalysts (200 mg), 90 °C, 4 h, O_2_ atmosphere. Error bars: 5%.

For practical industrial applications, the durability of catalysts under operating conditions is evaluated, especially when it comes to large-scale production [2,58]. The reusability of typical 2%Au_pre_/Ti_3_Al_x_C_2_T_y_ was examined in the two model reactions, and the test results were compared in Figure 6. The reaction rates were calculated on the basis of real Au loading. Because of the loss of spent catalysts after reaction (approximately 10%), additional fresh catalysts were supplemented to maintain an identical mass of catalysts for each run.

The results revealed that the reaction rates of HMF maintained similar in three runs and thereafter declined from 215.3 to 183.3 mmol·g_Au_^−1^·h^−1^, and the HMFCA selectivity declined from 90.3% to 78.3%. In the selective oxidation of Ph-CH_2_OH, the reaction rates of Ph-CH_2_OH exhibited gradual slight decline from 178.3 to 163.8 mmol·g_Au_^−1^·h^−1^, and the Ph-CHO selectivity declined from the fourth run (95.8%) to 87.8% in the fifth run. Various factors might also contribute to the deactivation, such as sintering of Au NPs, leaching of active sites, change in crystal structures which were difficult to identify based on the reusability test results. The ICP test of spent catalysts showed that the Au content was around 1.7% after five cycles in the selective oxidation of HMF, and was around 1.73% after use in the selective oxidation of benzyl alcohol, which were close to the original value (1.72%, Table S2, ESI).

The reasons for the identifiable decline in the last three runs were probably due to factors such as the carbon/hydrochar deposition that covered a proportion of active centers. As to HMF, the molecules are easy to form oligomers (e.g., dissolvable hydrochar species of humins [6]), the gradual accumulation of these byproducts occurs on the Au surface during repeated cycles, and progressively block the accessible active sites of Au NPs after the first two runs. Consequently, an observable reduction in reaction rate and HMFCA is observed in the subsequent three tests. In comparison, the reactant molecules of benzyl alcohol are relative stable as to form polymerized side products, and the molecules of benzaldehyde were easy to desorb; hence, the catalysts maintained more steady catalytic performances.

## 4. Conclusions

This work developed a facile synthesis strategy to prepare supported gold catalysts under one-pot hydrothermal synthetic conditions, utilizing the as-generated H_2_ during hydrothermal transformation of MAX phases in heated basic solutions to in situ reduce gold metal precursors. The strategy offers potentially viable large-scale preparation of supported gold catalysts for easy recycling from liquid-phase reactions, owing to the fibrous nature of Ti_3_Al_x_C_2_T_y_ supports; moreover, this strategy may also be applicable to design other supported metallic catalysts. More future work is required with regard to the substantial mechanistic insights into the Au metal–support interactions and their contributions to improving catalytic performances in selective oxidation HMF and aromatic alcohols. The newly developed series of catalysts Au_pre_/Ti_3_Al_x_C_2_T_y_ showed higher efficiency to load Au NPs, as compared with Au_dp_/Ti_3_Al_x_C_2_T_y_ prepared by the traditional deposition–precipitation method, thus enhancing the reaction rates of HMF and Ph-CH_2_OH. Moreover, the selectivity of HMFCA over 1%Au_pre_/Ti_3_Al_x_C_2_T_y_ (96.3%) was much higher than that over 1%Au_dp_/Ti_3_Al_x_C_2_T_y_ (84.3%). The reusability tests demonstrated that the typical Au_pre_/Ti_3_Al_x_C_2_T_y_ catalyst maintained reaction activity and product selectivity in at least three cycles, whereas the stability of Au_dp_/Ti_3_Al_x_C_2_T_y_ catalyst was relatively weaker in both model reactions.

## Figures and Tables

**Table 1:** Comparison of aromatic alcohols over the typical catalyst of 2%Au_pre_/Ti_3_Al_x_C_2_T_y._

Reagent	Target Product	Conversion/%	Selectivity/%
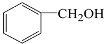 Benzyl alcohol	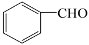 Benzaldehyde	70.1	>99
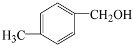 4-Methylbenzyl alcohol	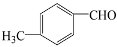 Methyl-benzaldehyde	68.0	>99
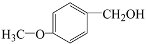 4-Methoxybenzyl alcohol	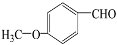 Methoxy-benzaldehyde	90.6	>99
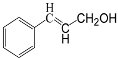 Cinnamic alcohol	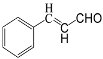 Cinnamaldehyde	40.2	>99

**Reaction conditions:** Catalyst (Au_pre_/Ti_3_Al_x_C_2_T_y_, 200 mg, the nominal loading of gold was 2%wt), aromatic alcohol (reactant, 3.5 mmol), sodium hydroxide (0.35 mmol), toluene (solven, 20 mL), O_2_ atmosphere, 4 h, 90 °C. The selectivity toward target product of aldehyde was higher than 99% for all reactions, and no other products were detectable.

## Data Availability

The original contributions presented in this study are included in the article/Supplementary Material. Further inquiries can be directed to the corresponding author.

## Supplementary Materials

The following supporting information can be downloaded at: https://www.mdpi.com/article/10.3390/ma19194179/s1, Figure S1: XRD patterns of (a) Au_pre_/Ti_3_Al_x_C_2_T_y_ and (b) Au_pre_/Ti_3_Al_x_C_2_T_y_ with different gold contents; Figure S2: High-resolution XPS analyses of key elements of C1s, O1s and Ti2p of Au_pre_/Ti_3_Al_x_C_2_T_y_ (T refers to –OH, ONa, or =O); Figure S3: The reaction rate in selective oxidation of (a) HMF to HMFCA and (b) Ph-CH_2_OH to Ph-CHO over Au_pre_/Ti_3_Al_x_C_2_T_y_ and Au_dp_/Ti_3_Al_x_C_2_T_y_ as the function of real Au loading. Reaction conditions: HMF (reactant, 1 mmol), H_2_O (solvent, 10 mL), Na_2_CO_3_ (2 mmol), catalysts (50 mg), 120 °C, 5 h, 0.5 MPa O_2,_ 600 rpm; andPh-CH_2_OH (reactant, 3.5 mmol), sodium hydroxide (0.35 mmol), toluene (solvent, 20 mL), catalysts (200 mg), 90 °C, 4 h, O_2_ atmosphere; Table S1: Summarized binding energies of Au^δ+^, Au and Au^δ-^ obtained from literature reports; Table S2: The real loading of gold on Au_pre_/Ti_3_Al_x_C_2_T_y_ was detected by ICP and shown below. Refs [59,60,61,62,63,64,65,66,67] are included in Supplementary Materials.
