# Supplementary material for: Facile In Situ Synthesis of Supported Gold Catalysts on Ti3AlC2-Based Nanofibers for Selective Oxidation of Biomass-Derived HMF and Aromatic Alcohols
Source: Materials (Basel). 2026 Sep 30;19(19):4179. doi: 10.3390/ma19194179 (PMC13666526; doi:10.3390/ma19194179)
Supplement: Supplementary file 1 [file materials-19-04179-s001.zip › materials-4573354-supplementary.pdf]

Electronic Supplementary Information

Facile In Situ Synthesis of Supported Gold Catalysts on Ti_3AlC_2-Based Nanofibers for Selective Oxidation of Biomass-Derived HMF and Aromatic Alcohols

Yicheng Sun,[1] Jun Chen,[2] Jun Yan,[3] and Xingguang Zhang[2,*]

Address:

[1] YK PAO School, 1800 Lane 900 North Sanxin Road, Songjiang District, Shanghai 201620, China

[2] School of Materials and Chemistry, University of Shanghai for Science and Technology, 516 Jungong Road, Shanghai 200093, China

[3] College of Chemical Engineering, Nanjing Forestry University, Nanjing 210037, China

* Correspondence: x.g.zhang@usst.edu.cn

Section S1. XRD patters of Au/$Ti_3Al_xC_2T_y$ with different gold contents

The crystal phases of all these catalysts were characterized by XRD patterns as shown in **Figure s1**, with different loadings of Au in theory: 0.25 wt%, 0.5 wt%, 1.0 wt%, 1.5 wt% and 2.0 wt%. The three peaks around 38.2°，44.5°，64.5° were the characteristic diffraction peaks of Au NPs, corresponding to the Au (111), Au (200) and Au (220) planes, respectively. And with the increase of gold content, these three peaks became more intense.

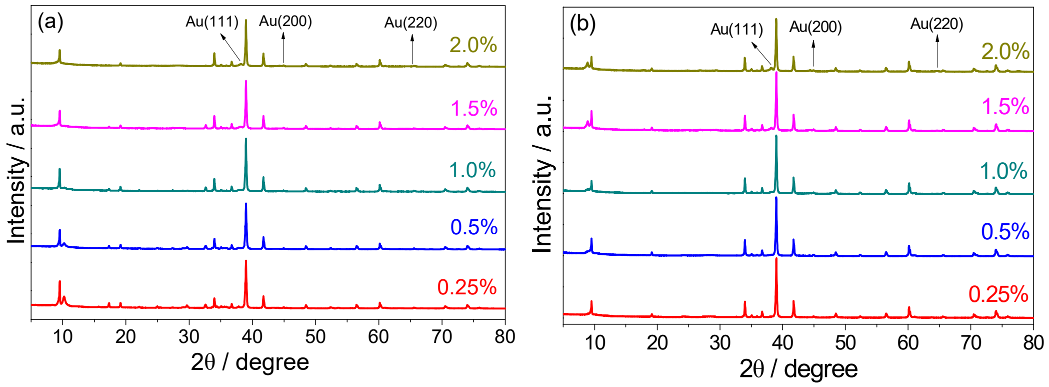

Figure s1 XRD patterns of **(a)** $Au_{pre}/Ti_3Al_xC_2T_y$ and **(b)** $Au_{pre}/Ti_3Al_xC_2T_y$ with different gold contents

Section S2. HR-XPS analysis of key elements

The HR-XPS analyses of other key elements were provided in **Figure s2**. The C 1s binding energy has the values of 284.8, 286.5, and 289.0 eV, corresponding to the bonds of C-C, C-O and C=O, respectively[1]. The O 1s spectra could be further deconvoluted into the major lattice O (Ti-O-Ti, 529.8 eV), surface O at bridging sites (Ti=O, 531.3 eV), surface O at top sites (Ti-OH/ONa, 532.5 eV) and O in adsorbed H_2O (534.0 eV) [2]. The Ti element showed two major spin-orbital peaks located at 458.1 eV and 463.8 eV are assigned to the $2p_{3/2}$ and $2p_{1/2}$ peaks of Ti^{4+} in $Ti_3Al_xC_2T_y$, moving to lower binding energies as compared with those in TiO_2 (458.7 eV for Ti2$p_{3/2}$ and 464.4 eV for Ti 2$p_{1/2}$), probably owing to the influence of substoichiometric titanium carbides (TiC_x, $x < 1$) or titanium oxycarbides (O-Ti-C) [3].

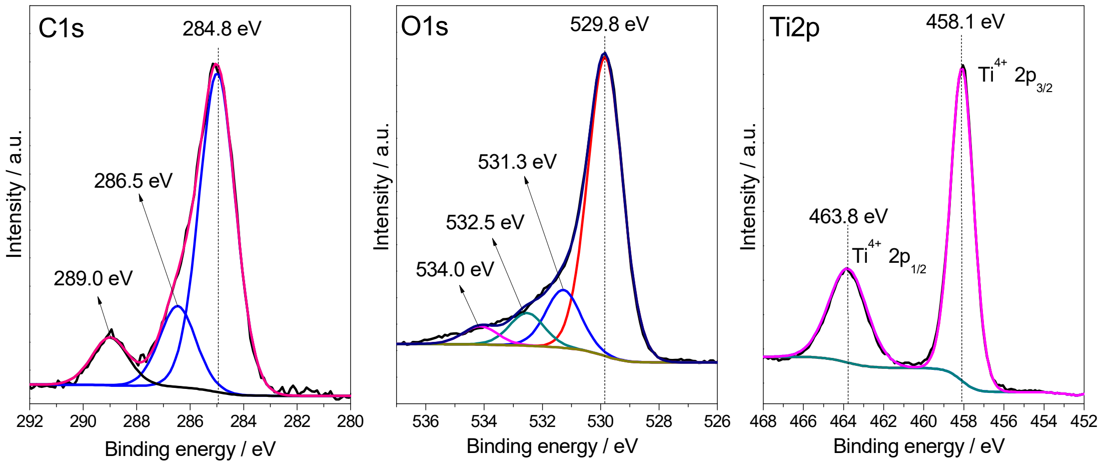

Figure s2 High-resolution XPS analyses of key elements of C1s, O1s and Ti2p of $Au_{pre}/Ti_3Al_xC_2T_y$ (T refers to –OH, ONa, or =O).

Table s1: Summarized binding energies of $Au^{\delta+}$, Au and $Au^{\delta-}$ obtained from literature reports.

Material	$Au^{\delta+}$ ($4f_{7/2}$) / eV	Au ($4f_{7/2}$) /eV	$Au^{\delta-}$ ($4f_{7/2}$) /eV	Ref
Au@TiO_{2-x}/ZnO	-	83.5	83.1	[4]
TiO_x@Au/TiO_2	-	83.5	83.1	[5]
Au@Ti/SiO_2-wcSMSI	-	83.8	83.4	[6]
Au-BiOCl	-	83.4	-	[7]
Au/TiC	-	84	-	[8]
Au/TiO_2-800	-	83.2	-	[9]
Au/HAP-800	-	83.8	-	[9]
Au/CeO_2	85.5 (Au^+)	83.8	-	[10]
Au/CeO_2	85.9 (Au^+)	84	-	[11]
Au-CeO_2@SBA-15	84.6 (Au^+)<br>86 (Au^{3+})	84	-	[12]
Au/FeO_x-TiO_2	86.3 (Au^{3+})	84	-	[13]

Note: -- refers to unavailable or undetectable

Section S3. ICP results

Table s2. The real loading of gold on Au_{pre}/$Ti_3Al_xC_2T_y$ was detected by ICP and shown below.

Nominal loading of Au		0.25 wt%	0.5 wt%	1.0 wt%	1.5 wt%	2.0 wt%
Real loading of Au	Au_{pre}	0.21	0.42	0.87	1.36	1.72
	Au_{dp}	0.19	0.26	0.54	1.07	1.34

Section S4. Reaction rates of HMF and benzyl alcohol

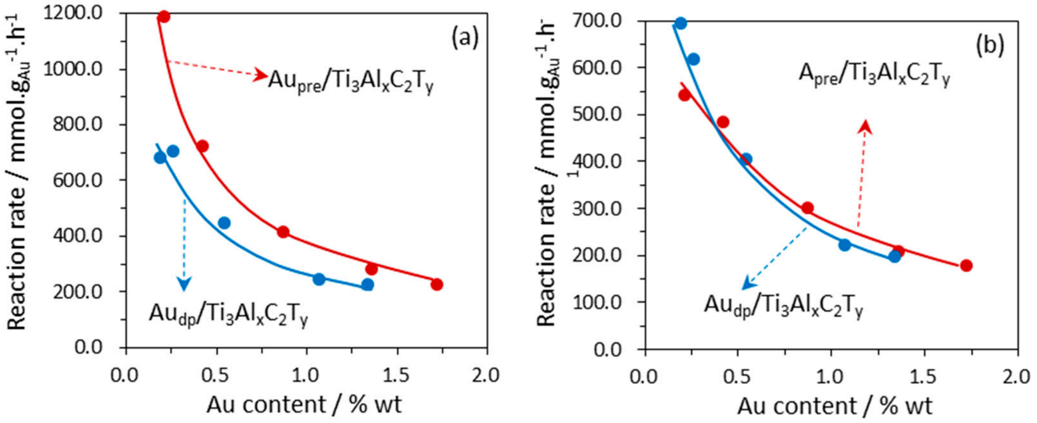

Figure s3. The reaction rate in selective oxidation of **(a)** HMF to HMFCA and **(b)** Ph-CH_2OH to Ph-CHO over $Au_{pre}/Ti_3Al_xC_2T_y$ and $Au_{dp}/Ti_3Al_xC_2T_y$ as the function of real Au loading. **Reaction conditions:** HMF (reactant, 1 mmol), H_2O (solvent, 10 mL), Na_2CO_3 (2 mmol), catalysts (50 mg),120 °C, 5 h, 0.5 MPa O_2, 600 rpm; and Ph-CH_2OH (reactant, 3.5 mmol), sodium hydroxide (0.35 mmol), toluene (solvent, 20 mL), catalysts (200 mg), 90°C, 4 h, O_2 atmosphere

References

[1] C. Peng, X. Yang, Y. Li, H. Yu, H. Wang, F. Peng, Hybrids of two-dimensional Ti_3C_2 and TiO_2 exposing {001} facets toward enhanced photocatalytic activity, ACS Appl. Mater. Interfaces 8(9) (2016) 6051-6060. https://doi.org/10.1021/acsami.5b11973.

[2] K. Wang, J. Lu, Y. Lu, C.H. Lau, Y. Zheng, X. Fan, Unravelling the C-C coupling in CO_2 photocatalytic reduction with H_2O on Au/TiO_{2-x}: Combination of plasmonic excitation and oxygen vacancy, Appl. Catal. B 292 (2021) 120147. https://doi.org/https://doi.org/10.1016/j.apcatb.2021.120147.

[3] X. Bao, H. Li, Z. Wang, F. Tong, M. Liu, Z. Zheng, P. Wang, H. Cheng, Y. Liu, Y. Dai, Y. Fan, Z. Li, B. Huang, TiO_2/Ti_3C_2 as an efficient photocatalyst for selective oxidation of benzyl alcohol to benzaldehyde, Appl. Catal. B 286 (2021) 119885. https://doi.org/https://doi.org/10.1016/j.apcatb.2021.119885.

[4] N. Liu, M. Xu, Y. Yang, S. Zhang, J. Zhang, W. Wang, L. Zheng, S. Hong, M. Wei, $Au^{\delta-}$–O_v–Ti^{3+} Interfacial site: catalytic active center toward low-temperature water gas shift reaction, ACS Catal. 9(4) (2019) 2707-2717. https://doi.org/10.1021/acscatal.8b04913.

[5] H.L. Tang, Y. Su, B.S. Zhang, A.F. Lee, M.A. Isaacs, K. Wilson, L. Li, Y.G. Ren, J.H. Huang, M. Haruta, B.T. Qiao, X. Liu, C.Z. Jin, D.S. Su, J.H. Wang, T. Zhang, Classical strong metal-support interactions between gold nanoparticles and titanium dioxide, Sci Adv 3(10) (2017) 1700231. https://doi.org/ARTN e1700231

[6] J. Zhang, H. Wang, L. Wang, S. Ali, C.T. Wang, L.X. Wang, X.J. Meng, B. Li, D.S. Su, F.S. Xiao, Wet-chemistry strong metal-support interactions in titania-supported Au catalysts, J. Am. Chem. Soc. 141(7) (2019) 2975-2983. https://doi.org/10.1021/jacs.8b10864.

[7] H. Li, F. Qin, Z. Yang, X. Cui, J. Wang, L. Zhang, New reaction pathway induced by plasmon for selective benzyl alcohol oxidation on BiOCl possessing oxygen vacancies, J. Am. Chem. Soc. 139(9) (2017) 3513-3521. https://doi.org/10.1021/jacs.6b12850.

[8] J.-H. Kim, H. Woo, J. Choi, H.-W. Jung, Y.-T. Kim, CO_2 electroreduction on Au/TiC: enhanced activity due to metal–support interaction, ACS Catal. 7(3) (2017) 2101-2106. https://doi.org/10.1021/acscatal.6b03706.

[9] H.L. Tang, F. Liu, J.K. Wei, B.T. Qiao, K.F. Zhao, Y. Su, C.Z. Jin, L. Li, J.Y. Liu, J.H. Wang, T. Zhang, Ultrastable hydroxyapatite/titanium-dioxide-supported gold nanocatalyst with strong metal-support interaction for carbon monoxide oxidation, Angew Chem Int Ed 55(36) (2016) 10606-10611. https://doi.org/10.1002/anie.201601823.

[10] X.-S. Huang, H. Sun, L.-C. Wang, Y.-M. Liu, K.-N. Fan, Y. Cao, Morphology effects of nanoscale ceria on the activity of Au/CeO_2 catalysts for low-temperature CO oxidation, Appl. Catal. B 90(1) (2009) 224-232. https://doi.org/https://doi.org/10.1016/j.apcatb.2009.03.015.

[11] R. Leppelt, B. Schumacher, V. Plzak, M. Kinne, R.J. Behm, Kinetics and mechanism of the low-temperature water–gas shift reaction on Au/CeO2 catalysts in an idealized reaction atmosphere, J. Catal. 244(2) (2006) 137-152. https://doi.org/https://doi.org/10.1016/j.jcat.2006.08.020.

[12] T. Wang, X. Yuan, S. Li, L. Zeng, J. Gong, CeO_2-modified Au@SBA-15 nanocatalysts for liquid-phase selective oxidation of benzyl alcohol, Nanoscale 7(17) (2015) 7593-7602. https://doi.org/10.1039/C5NR00246J.

[13] Y.-F. Yang, P. Sangeetha, Y.-W. Chen, Au/FeO_x−TiO_2 catalysts for the preferential oxidation of CO in a H_2 stream, Ind. Eng. Chem. Res. 48(23) (2009) 10402-10407. https://doi.org/10.1021/ie900806r.
